# *Lagochilascaris minor*: Susceptibility and Resistance to Experimental Infection in Mice Is Independent of H-2^a^ Haplotype and Correlates with the Immune Response in Immunized Animals

**DOI:** 10.1155/2010/610457

**Published:** 2010-06-22

**Authors:** Mônica Spadafora-Ferreira, Luciana Caetano Fernandes, Irmtraut Araci Hoffman Pfrimer, Cássia Regina Pichiteli, Denise Vilarinho Tambourgi, Ruy de Souza Lino-Junior, Mara Silvia Carvalhaes

**Affiliations:** ^1^Laboratory of Immunochemistry, Butantan Institute, 05503-900 São Paulo, Brazil; ^2^Laboratory of Immunology, State University of Goiás, 75132-903 Goiânia, Brazil; ^3^Laboratory of Immunology, Catholic University of Goiás, 74605-900 Goiânia, Brazil; ^4^Department of Microbiology, Immunology, Parasitology and Pathology, Institute of Tropical Pathology and Public Health, Federal University of Goiás, 74001-970 Goiânia, Brazil

## Abstract

Recently, we demonstrated that C57BL/6 mice are more susceptible to experimental lagochilascariosis than BALB/c mice. To investigate the pattern of infection and the role of the genetic background on susceptibility to infection, we studied experimental lagochilascariosis in H-2^a^ identical B10.A and A/J mice. Infected B10.A mice had a lower survival ratio and more severe lesions in the lungs than did A/J mice. Splenocytes of A/J mice immunized with the crude extract of the parasite showed increased proliferation and produced a higher level of interleukin 10 and interferon-*γ* in the presence of CE or concanavalin A when compared to B10.A mice. This suggests that resistance of A/J mice may be due to less severe lesions in lungs and other organs and a better immune response to parasite antigens. This paper provides evidence that major histocompatibility complex haplotype does not influence the survival to experimental infection with *L. minor*.

## 1. Introduction

Human lagochilascariosis is caused by *Lagochilascaris minor,* an infection that has been reported in individuals of both sexes. High-risk populations include those of lower socioeconomic status and rural residents. Lagochilascariosis is considered an emerging helminthosis described in the American continents and its diagnosis is underestimated [1]. The main clinical symptoms in humans are chronic lesions with abscess formation, usually affecting the neck and head tissues [2, 3]. Sometimes the parasite invades the pulmonary tissue and central nervous system, leading to death [4, 5]. Frequently, *L. minor* lesions contain multiple stages of the parasite (eggs, larvae, and adult worm), indicating autoinfection and favoring the development of chronic disease [2].

The natural life cycle of *L. minor* and its infection mechanism remain unknown. An experimental heteroxenous life cycle for the parasite has been described in mice and domestic cats [5, 6] as well as in wild rodents and cats [4]. Currently, mice are considered intermediate hosts for the parasite.

The capacity of *L. minor* to migrate across different human tissues is also observed in animal models of the disease [2]. In mice orally inoculated with *L. minor* infective eggs, hatched larvae can be observed migrating in the intestinal tract (6–12 h). After hatching, 3rd stage larvae (L3) migrate through intestinal mucosa into vessels and hepatic parenchyma (12 h) and disseminate to other tissues. Granulomatous lesions containing encysted L3 larvae have been found 30 days after infection in lungs, skeletal muscles, subcutaneous tissues, and lymph nodes [4]. In cats that eat carcasses of infected mice, L3 migrate from the stomach (6 h) to the upper portions of the digestive tract, where they develop into 4th-stage larvae (L4), reaching maturity after 12 days of infection. Adult worms located in the esophagus, pharynx and trachea, and rhino-oropharynx and cervical lymph nodes, can expel eggs that will be found in feces of infected cats [2].

We recently showed that BALB/c mice are more resistant to *L. minor* infection than C57BL/6 mice, having less severe lesions in the lungs, lower numbers of nodules with encysted larvae, fewer adult worms, and higher serum levels of IFN-*γ* [7]. The availability of isogenic strains of mice with different genetic backgrounds and the same H-2 haplotype has enabled the study of this host-parasite relationship, which is crucial to the establishment of susceptibility or resistance to infection. The manipulation of the host immune response system by *L. minor* antigens may be a key determinant of survival within the mammalian host. Therefore, the aims of this study were to evaluate infection in B10.A and A/J mice (both H-2^a^), compare the survival rate and tissue lesions, and analyze the proliferative response and production of IFN-*γ* and IL-10 in cultures of spleen cells from normal and immunized mice stimulated with the crude extract of the parasite (CE) or concanavalin A (ConA).

## 2. Materials and Methods

### 2.1. Mice

Six- to eight-week-old A/J and B10.A male mice were purchased from the University of São Paulo Animal Facility. They were given food and water “ad libitum” and handled according to local regulations. The Research Ethics Committee of the Federal University of Goiás approved the research protocols.

### 2.2. Parasites

Eggs from the parasite were collected from feces of *Felis domesticus* experimentally infected with a human isolate of *L. minor*. Feces from infected animals were subjected to Hoffman's method [8] and kept in culture in formalin solution (1%) at room temperature for 30 days. After the development of infective eggs containing third-stage (L3) larvae, cultures were submitted to Faust's method [9] for optimal recovery of eggs free from fecal debris [10]. Egg suspensions were washed five times (20 minutes/4.000 rpm) with phosphate-buffered saline (PBS, pH 7.4) and transferred to a graduated centrifuge tube. The eggs were counted on microscope slides. The final concentration was adjusted to 2 × 10^4^ eggs/mL and then used to infect the mice.

### 2.3. Experimental Infection Design and Survival Rate

In total, 60 A/J and 66 B10.A mice were orally inoculated with a suspension of 2 × 10^3^ ± 200  *L. minor* eggs per animal. In total, 20 A/J and 26 B10.A animals were followed for one year to determine survival rates. Forty animals were sacrificed (5 per day) at different time points (30, 45, 60, 90, 120, 150, 180, and 210 days postinfection) and submitted to necropsy for collection of organs for histopathology. Additionally, 40 A/J and 40 B10.A uninfected mice received saline orally and were used as follows: 20 animals served as controls for mortality (followed for one year), and 20 were necropsied and served as controls for histopathology (5 animals sacrificed at each time point: 30, 90, 150 and 210 days after infection).

### 2.4. Histopathological Analysis

Sections of spleen, lung, lymph node, liver, muscle, and subcutaneous nodules derived from groups of 5 uninfected and 5 infected B10.A and A/J mice were collected 30 to 210 days after infection, fixed in 10% neutral buffered formalin, embedded in paraffin and subsequently stained with hematoxylin and eosin (H & E) [11], Masson's trichrome for detection of fibrosis [12] and Lunas' staining [13] for characterization of eosinophils.

The granulomas were classified as primary or exudative granuloma, secondary or exudative-productive granuloma and tertiary or productive-fibrotic granuloma. The primary granuloma was characterized by an aggregation of macrophages surrounding the parasite. The secondary granuloma was characterized by a necrotic zone of variable extension, a cellular exudation zone, and presence of epithelioid cells. The tertiary granuloma was characterized by less necrotic cells and diminished cellular exudation with predominance of conjunctive tissue [14, 15].

### 2.5. Antigen Preparation and Immunization of Mice

*L. minor*-infected mice were euthanized 60–90 days postinfection. Encysted L3 larvae were collected from nodules under sterile conditions upon puncturing to allow spontaneous release of the parasite. After 20 washes in PBS, live L3 larvae were resuspended in PBS and sonicated in an IKA-TK8 disruptor. The crude extract (CE) of L3 larvae was then centrifuged at 50000 × g for 1 h at 4°C. The supernatant was collected and the protein content was estimated by the micro BCA method (Pierce, USA). Once aliquoted, the samples were stored at −80°C. Seven naive mice of each strain were immunized with 10 *μ*g of CE of *L. minor* in a volume of 0.1 mL. The control group (4 animals) received saline only. Mice were immunized subcutaneously four times at one-week intervals, and their spleens were collected one week after the final immunization for evaluation of lymphocyte proliferation and cytokine production.

### 2.6. Proliferation Assays

Spleens from A/J and B10.A mice were collected in RPMI medium supplemented with 2 mM L-glutamine, 1 mM sodium pyruvate, 100 U/mL penicillin, 100 *μ*g/mL streptomycin, 2 × 10^−5^ M 2-mercaptoethanol, 1% nonessential amino acids and 5% fetal calf serum (FCS) and were passed through nylon mesh to obtain spleen cells. After erythrocyte lyses, the cell suspension was washed twice in supplemented RPMI medium and plated in triplicate at 5 × 10^5^ cells/well of 96-well round-bottomed plates in a total volume of 200 *μ*l medium. Spleen cells were stimulated with 5 *μ*g/mL of ConA (Sigma) or 5 *μ*g/mL of CE of *L. minor*. This concentration of ConA and CE produced optimum responses in immunized animals when tested in within a range of 0.5 and 50 *μ*g/mL. Mitogen- and antigen-stimulated cultures were maintained for 5 days at 37°C and 5% CO_2_ in a humidified atmosphere. ^3^H-thymidine was added (0.5 *μ*Ci per well) for the last 18 hours of culture. Cells were then harvested on glass fiber filters, and the incorporated radioactivity was measured in a liquid scintillation counter. Data are indicated as counts per minute (cpm).

### 2.7. Cytokine Production

For analysis of secreted cytokines, 5 × 10^5^ spleen cells/well (as above) were plated in 96-well U-bottom tissue culture plates (Costar) and stimulated with ConA or CE (as above). After 48 hours, cell-free culture supernatants were collected and stored at −80°C until use. IL-10 and IFN*γ* were measured by a sandwich ELISA using the OpEIA kit (BD Bioscience, USA) according to the manufacturer's instructions. Cytokine analysis was performed based on a standard cytokine concentration curve with a detection limit of 15 pg/mL for both IL-10 and IFN*γ*. Results are expressed in pg/mL.

### 2.8. Statistical Analyses

Parasitological parameters and cytokines were expressed as mean ± standard error. Data from the two groups were analyzed using the Mann-Whitney *U* test. The survival curve was analyzed using the Kaplan and Meier [16] method, and the differences between groups were tested using the Log-rank test.

## 3. Results

### 3.1. Survival Rate of Mice Infected with Lagochilascaris Minor

**Figure 1:**
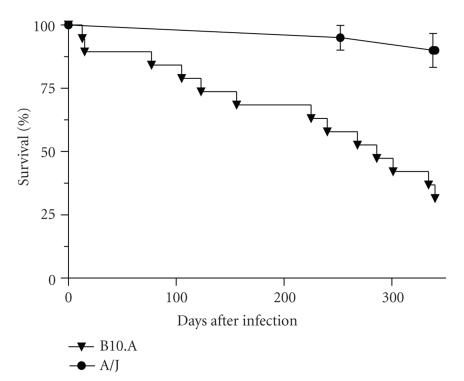
Cumulative survival rates during the infection period of A/J (•) and B10.A (▾) mice, each orally inoculated with 2000 viable eggs of *Lagochilascaris minor*. The survival ratio was determined using the Kaplan-Meier test (*P* = .0003).

B10.A mice began to die on day 13 postinfection, reaching only 26% survival on day 340, with a median survival time of 246.5 days. In contrast, A/J mice began to die later, on day 252 of infection, reaching 90% survival on day 340 (Figure 1). A/J mice displayed significantly greater survival rates than B10.A throughout the entire period of infection (*P* = .0003). In the control group, only two B10.A mice and one A/J mouse died by day 340 postinfection.

### 3.2. Histopathological Findings

**Figure 2:**
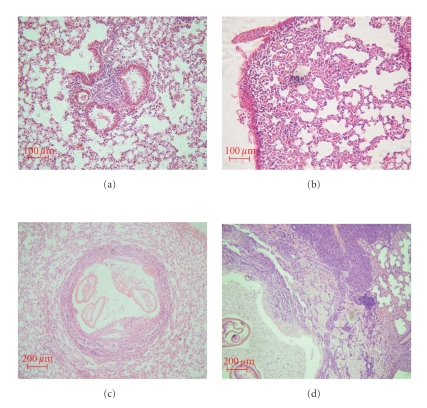
Lungs of mice infected with *Lagochilascaris minor*. Lungs of (a) an A/J mouse with perivascular and peribronchial mild inflammatory infiltration of macrophages and a few neutrophils (H&E, scale = 100 *μ*m) and (b) a B10.A mouse (b) with severe and diffuse inflammatory infiltration composed of macrophages, neutrophils and few eosinophils (H&E, scale = 100 *μ*m), both collected 30 days after infection. Lungs of (c) A/J mouse with varying degrees of damage, including secondary granuloma containing larvae in different degrees of destruction, concentric fibrosis and foci of inflammatory infiltration composed of macrophages (H&E, scale = 200 *μ*m) and (d) B10.A mouse with secondary granuloma and severe and diffuse inflammatory infiltration in the lungs with a predominance of macrophages and few neutrophils (H&E, scale = 200 *μ*m), both collected 60 days after infection.

In the initial infection phase (0 to 30 days postinfection), A/J mice presented a primary granuloma in the lungs containing preserved larvae, perivascular and peribronchial moderate inflammatory infiltration of macrophages and discrete infiltration of neutrophils (Figure 2(a)). In the intermediate infection phase (45 to 90 days postinfection), a secondary granuloma was observed with severe and diffuse inflammatory infiltration of foaming macrophages, multinuclear giant cells and a small number of eosinophils and fibroblasts in the lungs (Figure 2(c)). In late infection phase (120 days postinfection), a tertiary granuloma was observed in the lungs, containing disrupted third stage larvae with moderate necrosis and destruction of the tissue, concentric fibrosis, and inflammatory infiltration foci composed of macrophages. 

In contrast, B10A mice in the initial phase of the infection (0 to 30 days postinfection), presented severe and diffuse inflammatory infiltration in the lungs, composed of macrophages, neutrophils and small number of eosinophils (Figure 2(b)). In the intermediate phase (45 to 90 days postinfection), a secondary granuloma with severe and diffuse inflammatory infiltration was observed in the lungs with a predominance of macrophages and a small number of neutrophils (Figure 2(d)). In late-phase infection (120 days postinfection) tertiary granuloma with concentric fibrosis intercalated by foaming macrophages and multinuclear giant cells was observed in the lungs. The moderate inflammatory infiltration of the lungs was diffuse and composed of macrophages and few neutrophils.

In both mouse lineages, the liver developed large hepatocytes with bilobular nuclei and congestion with occasional perivascular inflammatory infiltrate. The spleen and lymph nodes had lymphoid follicles with expanded germinative centers. Granulomas were also found in adipose tissue, skeletal musculature, and lymph nodes.

### 3.3. Proliferative Response of Spleen Cells

**Figure 3:**
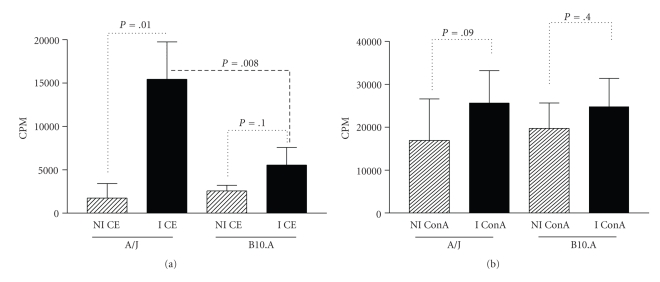
Proliferative response of spleen cells from (a) A/J and (b) B10.A mice immunized with crude extract (CE) of L3 larvae from *Lagochilascaris minor* stimulated in vitro with ConA or CE. The negative control is the [^3^H-thymidine] uptake of cells cultivated without stimulation. Bars represent mean values ± SD.

Spleen cells from A/J mice immunized with the crude extract of the parasite showed higher levels of proliferation in the presence of CE (*P* = .01) when compared to nonimmunized controls (Figures 3(a) and 3(b)). A/J immunized mice also exhibited a greater proliferative response against CE antigen compared to B10.A immunized mice (*P* = .008).

In addition, spleen cells of A/J and B10.A mice immunized with the crude extract (CE) of the parasite showed higher levels of proliferation in the presence of ConA when compared to nonimmunized mice, although the difference was not statistically significant.

### 3.4. Production of IL-10 and IFN-*γ*

**Figure 4:**
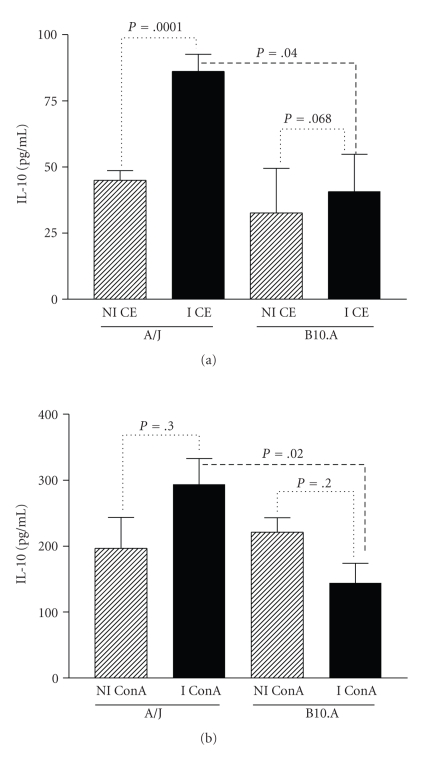
Level of IL-10 in the culture supernatant of spleen cells from A/J and B10.A mice nonimmunized (NI) and immunized (I) with the crude extract of L3 larvae of *Lagochilascaris minor*, stimulated with (a) ConA or (b) CE. Spleen cells were stimulated in vitro, and the supernatant collected after 48 hours. Cytokine production was considered positive if over the detection limit of 15 pg/mL for IL-10. Bars represent the mean values ± SD.

Spleen cells from A/J immunized mice produced higher levels of IL-10 induced by CE than did nonimmunized mice, but in the B10.A strain, the difference in IL-10 production was not statistically significant (Figure 4(a)). Additionally, A/J immunized mice produced more IL-10 against CE antigen compared to B10.A immunized mice (*P* = .04).

 In A/J immunized mice, the IL-10 production by spleen cells stimulated with ConA was higher than that of nonimmunized animals, although the results were not statistically significant (Figure 4(b)). In contrast, B10.A immunized mice produced lower amounts of IL-10 when compared to nonimmunized animals, but these results were also not statistically significant. However, IL-10 production induced by ConA stimulation of spleen cells from A/J immunized mice was significantly higher than the amounts produced by B10.A mice (*P* = .02). 

**Figure 5:**
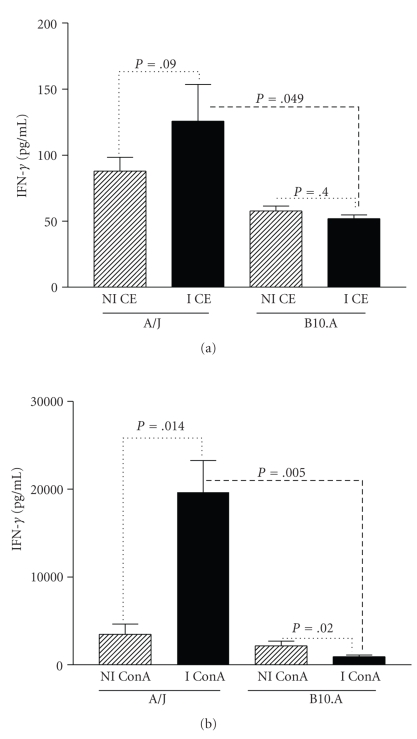
Level of IFN*γ* in the culture supernatant of spleen cells from A/J and B10.A mice nonimmunized (NI) and immunized (I) with the crude extract of L3 larvae of *Lagochilascaris minor*, stimulated with (a) ConA or (b) CE. Spleen cells were stimulated in vitro and the supernatant was collected after 48 hours. Cytokine production was considered positive if over the detection limit of 15 pg/mL for IFN*γ*. Bars represent the mean values ± SD.

Levels of IFN-*γ* induced by CE in spleen cells of A/J immunized mice were higher than nonimmunized mice, although not statistically different. IFN-*γ* production induced by CE stimulation of spleen cells from B10.A immunized mice was similar to from nonimmunized animals. Moreover, the IFN-*γ* production induced by CE stimulation of spleen cells from A/J immunized mice was higher compared to B10.A mice (*P* = .049) (Figure 5(a)).

In addition, levels of IFN-*γ* produced by spleen cells of immunized A/J mice stimulated with ConA were higher than those presented by nonimmunized mice (*P* = .014). In contrast, spleen cells of B10.A immunized mice produced lower levels of IFN-*γ* induced by ConA than did nonimmunized control animals (*P* = .02). The IFN-*γ* production induced by ConA stimulation of spleen cells from A/J immunized mice was also higher than that of B10.A mice (*P* = .005) (Figure 5(b)).

## 4. Discussion

This paper provides evidence that the MHC complex (H-2^a^ haplotype) of mice infected with *L. minor* does not influence survival during experimental infection. The survival rates of infected A/J mice were significantly higher than those of infected B10.A mice, although these isogenic mouse strains have different genetic backgrounds and the same H-2 haplotype (H-2^a^). H-2 is an important factor, but we must consider that susceptibility to parasitic infection is polygenic.

In fact, host genetic factors have a major influence on the susceptibility of mammals to infection by a variety of microorganism [17]. The genetically determined differences between individuals in a population affect the efficiency of the immune response, and thus the host phenotype of susceptibility. In experimental paracoccidioidomycosis, B10.A mice behave as susceptible and A/J as resistant strain to fungal infection, that correlates with differences in the immune response, since resistant mice showed prevalent type 1 immunity whereas susceptible B10.A presented a progressive form of infection with impaired IFN-*γ* secretion [18, 19]. Acquired immunity against *Leishmania donovani* has been shown to be under the control of genes within the H-2 locus: in a B10 genetic background, mice carrying the H-2^b^ haplotype present a cure phenotype; whereas, the H-2^d^ haplotype strains developed a noncure disease, in a manner dependent of the cytokines produced [20]. The influence of the H-2 linked as wells as non-H-2 linked genes on the immune response was also shown by the infection of mice with *Trichinella spirallis,* which affects the expulsion of the nematode from inbred and congenic mice [21, 22]. 

The lesions detected in infected B10.A mice were similar to those described by Semerene et al. [23] in C57BL/6 mice. Severe perivasculitis, vasculitis, and interstitial inflammation were visualized in the lungs, in the early phase of infection. We also observed organized granulomas around intact L3/L4 larvae with concentric fibrosis, macrophages, neutrophils, and small number of eosinophils in the lungs of these mice, in the intermediate phase of the infection (45–90 days after infection). In contrast, the lungs of infected A/J mice had moderate perivasculitis, vasculitis, and interstitial inflammation, in the early phase of the infection, while during the intermediate phase of infection we detected less severe organized granulomas around intact or disrupted L3/L4 larvae with discrete fibrosis and presence of macrophages, plasma cells, few neutrophils and rare eosinophils, These results indicate that pulmonary lesions are of greater severity in the B10.A strain and correlate with mortality.

The differences noted in mouse susceptibility to *L. minor* infection with distinct survival rates and tissue lesions may also be influenced by individual host immune response to the parasite. Different cell populations are involved in the immune response to the parasite: T and B cells, and mononuclear phagocytes. Th1 cells produce IFN-*γ* and TNF and mediate the immune response against intracellular parasites. Th2 cells produce IL-4, IL-5, and IL-13, which stimulate humoral responses and are known to be involved in resistance to extracellular helminths such as *Nipostrongylus brasiliensis *[24]. Antibodies to *L. minor* (IgG, IgM and IgA) were detected in higher levels in A/J infected mice, when compared to B10.A infected mice, and it may reflect its greater resistance to infection [25]. The recently described Th17 cells that produce IL-17 play a key role in inflammation and the activation of neutrophils and immunity to microorganisms, particularly at mucosal surfaces. Regulatory T cells (Tr) of different subtypes produce IL-10 and/or TGF-*β*, and can regulate Th1, Th2 and Th17 cells, B cells, and macrophages [26–28]. IFN-*γ* is the signature cytokine produced by Th1 cells, but can be also derived from Tc, NK cells, and B cells in small proportion. IL-10 is the major cytokine produced by Tr cells, but can be also derived from B cells and monocytes [29, 30]. 

Considering that the participation of these different cell populations and the manipulation of the host immune response system by *L. minor* antigens may be a key determinant to their survival within a mammalian host, we immunized mice with the CE parasite antigen and analyzed the immune response.

In this paper, we investigated the proliferative response and cytokine production of spleen cells from mice immunized with the CE of *L. minor* L3 larvae. Spleen lymphocytes from A/J mice immunized with CE and stimulated with ConA (positive control) or CE of the parasite showed better proliferative response when compared to B10.A mice, particularly against the CE of the parasite. CE is composed of intracellular and membrane components of the parasite that may stimulate specific spleen cells from A/J mice better than B10.A mice. 

Immunized A/J mice showed significantly increased production of IL-10 against ConA and CE. On the other hand, spleen cells of immunized B10.A produced less IL-10 induced by CE antigens. Spleen cells from immunized A/J mice produced more IFN-*γ* when stimulated with ConA or CE than did spleen cells from B10.A immunized mice. Moreover, immunized B10.A mice produced less IFN-*γ* when stimulated with ConA when compared to nonimmunized controls. 

In conclusion, the A/J mice, which are more resistant to infection, displayed a better proliferative response and greater production of IL-10 and IFN-*γ* against CE antigen and ConA when immunized with *L. minor* antigens, compared to the susceptible B10.A mice which presented a lower proliferative response and less IL-10 and IFN-*γ*.

This intense inflammatory response may be a consequence of an uncontrolled reaction caused by the small amount of IL-10 produced in B10.A mice. In A/J mice, the concomitant production of IL-10 and IFN*γ* may control the tissue lesions and the number of parasites, limiting collateral tissue damage caused by a robust antiparasitic immune response. In fact, when we compared experimental lagochilascariosis in C57BL/6 and BALB/c mice, the latter were more resistant to infection and produced more IFN*γ* and IL-10 [7]. 

The high mortality observed in B10.A infected mice may be a consequence of the intense inflammatory reaction without control by regulatory cytokines. We are currently investigating the kinetics of cytokine production in A/J and B10.A mice infected with *L. minor* to better understand the immunological mechanisms involved in the resistance to the infection.
